# Comorbidity issues in the pharmacological treatment of pathological gambling: a critical review

**DOI:** 10.1186/1745-0179-1-21

**Published:** 2005-10-10

**Authors:** Bernardo Dell'Osso, Andrea Allen, Eric Hollander

**Affiliations:** 1 Compulsive, Impulsive and Anxiety Disorders Program, Department of Psychiatry, Mount Sinai School of Medicine, One Gustave L. Levy Place, New York, NY 10029, USA; 2 Compulsive, Impulsive and Anxiety Disorders Program, Department of Psychiatry, Mount Sinai School of Medicine, One Gustave L. Levy Place, New York, NY 10029, USA; 3 Compulsive, Impulsive and Anxiety Disorders Program, Department of Psychiatry, Mount Sinai School of Medicine, One Gustave L. Levy Place, New York, NY 10029, USA

**Keywords:** Pathological Gambling, Impulse Control Disorders, Comorbidity, Compulsive-Impulsive Spectrum, Subthreshold symptoms

## Abstract

**Background:**

Pathological Gambling (PG) is an impulse control disorder often comorbid with other psychopathology, particularly bipolar spectrum disorders, attention deficit/hyperactivity disorder, obsessive-compulsive disorder (OCD) and substance abuse. This paper reviews the published literature on the pharmacological management of PG, highlighting how clinical and subclinical comorbid psychopathology influences the choice of pharmacological treatment.

**Methods:**

Using Medline, the authors reviewed relevant articles published on this topic from1995 to 2005, focusing on the best-designed studies for inclusion.

**Results:**

Much of the literature on PG-treatment presupposes different theories regarding this disorder. Data suggest the utility of differentiating the pharmacotherapy of pathological gamblers in light of their comorbid profile, specifically assessing for comorbid bipolar, ADHD, OCD, and substance abuse disorders.

**Conclusion:**

Decisions about pharmacological treatment of PG should take into account current and previous comorbid disorders which influence treatment selection.

## Background

Pathological gambling (PG) is an impulse control disorder not otherwise specified (ICD-NOS) [1] that is characterized by recurrent and maladaptive patterns of gambling behavior and significantly disrupts the patient's functioning in the personal, familial, or vocational spheres. It is assumed to be a chronic disorder, with a clinical course that is continuous, unremitting, or episodic [2]. Its prevalence ranges from 1% to 3% of the US adult population [3,4], and there has been a dramatic increase in PG over the last decade, due to the legalization and availability of new forms of gambling in most Western countries. Despite a prevalence even higher than that of schizophrenia or bipolar disorder, little is known regarding effective treatments, particularly pharmacotherapies, for PG. In addition, currently, no medications have been approved by the U.S. Food and Drug Administration for the treatment of this impairing and common disorder.

A crucial issue to consider in approaching PG is represented by the high rates of comorbidity among pathological gamblers. The majority of these patients, at least those seeking treatment, have been found to score significantly higher than control populations on measures of depression [5], and have shown high incidences of various psychiatric disorders, including bipolar, anxiety and substance use disorders [6]. This frequent comorbidity is not surprising if we focus on the psychopathological core features of PG: impulsivity, compulsive drive to gamble, addictive features such as withdrawal symptoms during gambling abstinence, and bipolar features such as urges, pleasure seeking and decreased judgment due to unrealistic appraisal of the individuals' own abilities. Several authors have associated some of these core features to neurobiological data and clinical aspects of treatment-response, and have conceptualized PG as belonging to different spectrum classification models, in which the main psychiatric disorders of reference are obsessive-compulsive disorder (OCD), addictive disorders, and affective disorders. These models of categorization provide the basis and rationale for the use of specific pharmacological treatments in pathological gamblers. In addition, they may also suggest, according to consistent findings reported in some trials, the presence of specific subgroups of patients with similar core features, comorbid profiles and treatment-responses within the population of pathological gamblers.

### Main classification models for Pathological Gambling

The traditional nosographic model includes PG within ICDs-NOS; evidence supporting this categorization is the elevated rates of comorbidity between these disorders, and the similarity in phenomenology between PG and other ICDs. These similarities include the temptation to perform some behavior notwithstanding its detrimental consequences for the person, a growing emotional tension before performing the act, a gratifying feeling while performing the behavior, and sometimes, a feeling of guilt following the behavior. However, in addition to this classification model, at least three other conceptualizations have been historically proposed for the classification of PG [7].

PG has also been conceptualized as an obsessive-compulsive (OC) spectrum disorder, within the impulsive cluster [8]. Patients with OC spectrum disorders, in fact, experience unpleasant feelings and physiological activation that result in an intense desire to perform a specific behavior in order to relieve the unpleasant feelings [9-11]; this is the case in PG. In addition, a reduced capacity to resist gambling thoughts would lead to excessive gambling, in particular in the advanced phases of the disorder [12]. However, these patients differ from patients with OCD in important ways. Gambling behavior and thoughts are often experienced by these patients as ego-syntonic, while OCD obsessions and compulsions are generally ego-dystonic. In addition, the excessive doubt, frequently experienced by OCD patients [10,13,14] as well as their compulsions, characterized by harm avoidance, risk aversion and anticipatory anxiety [14], are not characteristic of pathological gamblers. OC spectrum disorders do differ along the dimension of risk aversion vs risk taking; the compulsive disorders are characterized by an overestimation of harm and by risk aversion while the impulsive disorders are characterized by an underestimation of risk and by risk seeking.

PG has occasionally been characterized as an affective spectrum disorder. Notwithstanding the high rates of comorbidity between depression and PG [15-18] and the frequent presence of suicidality and suicidal ideation among these patients [19-22], the link between these two disorders has been questioned by several authors. With regard to suicidality, for example, is possible to guess that high levels of impulsivity, often present in pathological gamblers, may lead to suicidality independently from depression. On the other hand, data from several studies suggest that PG is a bipolar spectrum disorder and, considering that the comorbidity between these two disorders is estimated to be approximately 24% [23], have led some authors to the conclusion that impulsivity and bipolarity are related [24]. Other authors have stressed that certain PG core features resemble some characteristic aspects of bipolar spectrum disorders. The experience of the urge in PG, for example, may represent a potential overlap between pathological gamblers and patients with bipolar spectrum disorders. The pleasure seeking and impaired judgment resulting from an unrealistic evaluation of one's own abilities that are seen in pathological gamblers resemble characteristics seen in individuals with bipolar disorder [25]. Both disorders involve potentially harmful but pleasurable behavior and acting without forethought. Finally, these disorders generally have their onset in early adulthood and have an episodic course.

Another interesting model proposed for PG is as a non-substance addiction. PG and substance use disorders, in fact, share several features. Important features common to these mental disorders include the intense desire to satisfy a need; the lack of control over the substance use or behavior; certain aspects of abstinence and tolerance; the obsessive thoughts about the substance use or the activity; and the continuous engagement in the behavior despite experiencing social and occupational consequences [26]. For example, pathological gamblers often increase the frequency of their bets or the amount of money gambled in order to achieve the desired level of excitement and this behavior is suggestive of drug tolerance. Finally, high comorbidity rates between PG and substance use disorders [23,27-33] particularly alcohol and nicotine abuse and dependence, give further support to this model.

Being able to explain PG using different models does not necessarily imply that these models are incompatible; rather it may suggest the possible presence of subtypes of pathological gamblers. Being able to identify different subtypes that share similar phenomenological and clinical characteristics might not only lead to a better understanding of this disorder, but better use of pharmacological options. In fact, if it is possible to distinguish different clinical subtypes, it would seem logical that pharmacological treatments could be optimized to fit specific subgroups. Several core symptoms of PG could conceivably be targeted for treatment: impulsive symptoms, compulsive symptoms, bipolar symptoms and addictive symptoms. A rational pharmacological choice would take into account which of these symptom domains seem to dominate, as well as a patient's comorbid disorders, because treatment should ultimately target all clinically significant symptoms in the individual patient. Actually, the ability of specific pharmacological treatments to improve gambling behavior in some patients and the lack of efficacy or, even the worsening, in some other patients, suggests the presence of specific subtypes of pathological gamblers and supports the possibility of targeting pharmacotherapy based on the patient's unique symptoms and comorbidities.

## Methods

In this review we used a Medline search to locate the published articles from 1995 to 2005. The most weight was given to double blind, placebo-controlled studies due to the high placebo response rates reported in most trials; open-label studies and case-reports have also been included. In addition, we focused on studies that were relevant to understanding the connection between the characteristics of the samples, the comorbidity profiles, the drug administrated and the clinical response achieved.

The variety of drugs employed in the treatment of PG includes serotonin reuptake inhibitors (SRIs) and other antidepressants, mood stabilizers, and opioid antagonists. We did not include the atypical antipsychotics since there has been little systematic investigation of them in PG, and there is little evidence of their effectiveness in this disorder.

## Results

### Serotonin Reuptake Inhibitors (see Tables 1, 2 - additional file: 1)

Serotonin reuptake inhibitors, considered first-line treatment for OCD [34], have been shown to be effective in treating impulsivity in other ICDs and OC spectrum disorders [35-39]. Furthermore, neurobiological data indicating serotonergic dysfunction in PG [40-42], a phenomenological link to compulsivity [8], and a possible response in PG to the SRI clomipramine [43], have given further support for the use of the SRIs in this disorder. To date, the following SRI compounds have been tested for the treatment of PG: fluvoxamine, paroxetine, citalopram and clomipramine.

#### Fluvoxamine

The possible efficacy of fluvoxamine in the treatment of PG has been tested by three randomized, placebo-controlled studies. In an initial pilot study conducted by our group [44], 16 pathological gamblers were enrolled in a single-blind, 16-week, crossover study at the end of which 7 of the 10 completers receiving fluvoxamine were found to be treatment responders with notable improvements on the main outcome measures: the Pathological Gambling Yale-Brown Obsessive Compulsive Scale (PG-YBOCS, reduction ≥ 25%)[45] and the Clinical Global Impressions Scale[46] (CGI score 2, very much improved or 1, much improved). The mean fluvoxamine dose at the end of the study was 207 mg/day and the 7 treatment responders reported a total abstinence from gambling behavior. It is noteworthy that among the 3 fluvoxamine non-responders, 2 patients had a history of cyclothymia, the mildest form of bipolar disorder, which raised the possibility of a symptomatologic exacerbation with this SRI as well as a gambling relapse.

In a subsequent double-blind crossover study [47] comparing fluvoxamine to placebo, a group of 10 male patients completed the 16-week trial. The percent improvement on the CGI-PG was significantly greater for fluvoxamine (40.6%) than for placebo (16.6%), and the percent improvement on PG-YBOCS, although not statistically significant, was greater for the group treated with fluvoxamine (33.4%) than with placebo (28%). The average fluvoxamine dosage at the end point was 195 mg/day. In this study, however, the patient group was limited and relatively homogenous, excluding gamblers with current comorbid drug or alcohol abuse as well as bipolar I and II patients.

In another double-blind, parallel, placebo-controlled trial [48], 13 pathological gamblers of the 32 enrolled completed a 6 month-study, receiving a mean fluvoxamine dosage of 200 mg/ day at endpoint. Blanco and colleagues, however, reported a statistically significant improvement with fluvoxamine compared with placebo only for males and for young pathological gamblers. Furthermore, a high drop-out rate in the fluvoxamine group, a high rate of placebo response (59%) and the concomitant psychotherapy received by some patients complicate the interpretation of the results. We cannot rule out that, notwithstanding the absence of another current Axis I disorder in the sample, the presence of subthreshold comorbid psychopathology might have decreased the homogeneity of the sample, suggesting different subtypes of pathological gamblers.

Taken as a whole, fluvoxamine trials support the efficacy of this compound in PG, although in some cases the efficacy was not statistically significant, as compared with placebo. Drug dosages ranged between 100 and 250 mg/day. It's noteworthy that at least in the first study different patterns of treatment-response were related to different comorbidity profiles.

#### Paroxetine

In a study conducted by Kim and colleagues, 45 patients were randomized to paroxetine up to 60 mg/day or to placebo in a double-blind, 8-week trial [49]. At endpoint, there were greater, and statistically significant, improvements (G-SAS [50], p = .042 and CGI, p = .025) in the paroxetine group than in the placebo group. As the authors reported, all subjects had low baseline scores at the Hamilton Depression Rating Scale (HDRS) [51] and Hamilton Anxiety Rating Scale (HARS) [52] suggesting low comorbidity rates in the sample for depression and anxiety. In addition, patients with other Axis I disorders were excluded from the study. However, it is possible to assume that the low presence of comorbidity might in turn be indicative of a specific subgroup of gamblers particularly sensitive to the SRI treatment.

In a later multicenter, double-blind, placebo-controlled trial [53], Grant and co-workers randomly assigned 76 pathological gamblers to paroxetine, up to 60 mg/day, or to placebo for 16 weeks. A total of 45 patients completed the study. The paroxetine group showed a greater percentage of responders (59%) at each study visit, compared with placebo (49%), but failed to demonstrate statistical significance over placebo on the outcome measures (PG-CGI scores of 1 or 2). Current Axis I disorders as well as a past history of bipolar, psychotic, alcohol or substance use disorders were exclusion criteria. The results of this study did not replicate the previous findings by Kim and colleagues, and found a notable placebo response for pathological gamblers without relevant comorbid conditions. Grant and colleagues suggested that a possible interpretation of these results might be the tendency to reduce unwanted behaviors when greater attention is focused on them, which might also increase motivation. However, the presence of subthreshold psychopathology was not investigated and whether or not this lack of response was related to particular subthreshold comorbid conditions.

#### Citalopram

In the only open-label study performed with citalopram [54], Zimmerman and colleagues reported a clinically significant improvement (PG-CGI scores of 1 or 2) for 13 of the 15 patients participating in the trial. The final mean dosage of citalopram was approximately 35 mg/day. Given the design of the study, which did not control for placebo response, it is not possible to calculate the actual effect of citalopram. However, it is noteworthy that while psychotic disorders, mania and hypomania, as well as drug or alcohol dependence were excluded, depression, anxiety, eating or impulse-control disorders were not. This difference could be important in the understanding the impressive clinical response in this study, suggesting that these particular comorbid disorders might not represent a specific contraindication to the use of the SSRIs

### Other antidepressants (see Table 3 - additional file: 1)

#### Nefazodone

In 2002, Pallanti and our group enrolled 14 patients with PG in an 8-week open-label oral nefazodone trial [55]. The sample included other Axis I comorbid disorders such as bipolar II, cyclothymia, depression, panic disorder and social phobia. At the endpoint, 9 of the 12 completers were classified as responders (reduction ≥ 25% PG-Y-BOCS and score of 1 or 2 on PG-CGI). The mean endpoint dosage of nefazodone was 350 mg/ day. This phenylpiperazine antidepressant with 5HT_2 _receptor antagonist properties and mixed noradrenergic/serotonergic reuptake inhibitor effects showed a good response profile in this group of pathological gamblers with other comorbid psychopathology. However, the study design as well as the limited size of the sample does not allow definitive conclusions.

#### Dopamine Reuptake Inhibitors

More recently, Black reported positive findings in an open-label trial [56] involving 10 pathological gamblers treated with bupropion up to 300 mg/day for 8 weeks. All subjects reported improvement (reduction from 20.3 to 8.8 on the PG-Y-BOCS and score of 1 or 2 on PG-CGI). Given patients' initial mild to moderate levels of ADHD traits, the author suggested that bupropion might reduce impulsiveness and improve attention span. In addition, he hypothesized that pathological gamblers with comorbid ADHD might represent a distinct subgroup of patients requiring a specific treatment.

### Opioid Antagonists (see Table 4 - additional file: 1)

Naltrexone, a long-acting opioid antagonist, is the only compound in this category that has been found to be effective in the treatment of PG. This compound blocks the effect of endogenous endorphins on central opiate receptors and also inhibits dopamine release in the nucleus accumbens, acting on neuronal pathways involved in reward, pleasure and urge. The inhibition of dopamine release in the nucleus accumbens, through the disinhibition of γ-aminobutyric acid (GABA) input to the dopamine neurons in the ventral tegmental area [57-66], is one of the most consistent reasons given for the use of naltrexone in ICDs. Another important pharmacological action of naltrexone in the central nervous system is the antagonism of the μ-opioid receptor, which is the site of action of beta-endorphins, morphine and heroin. Shinohara and colleagues [67] have suggested a possible role of this system in the physiological responses to gambling, given its involvement in the processing of reward, pleasure and pain. In addition, some neurobiological and neuroimaging reports [68,69] would confirm the relationship between some specific brain areas (nucleus accumbens, orbitofrontal cortex and motor limbic system), in which naltrexone is supposed to act, and their physiological role in the processes of reward and urge, typically abnormal in pathological gamblers.

So far, the clinical use of naltrexone has shown mixed results in treating urge related disorders such as alcohol dependence, OCD, bulimia nervosa, kleptomania, and self-injurious behaviors [70-74]. In a case report [70] of an open label treatment, Crockford and el-Guebaly reported that naltrexone at 50 mg/ day was effective in a patient suffering from both PG and alcohol dependence. In another case-report [75], a 55-year-old man with PG and compulsive shopping markedly improved while taking 100 mg/day of naltrexone. In a 6-week open-label study [76], 17 pathological gamblers without severe psychiatric comorbidity showed a statistically significant reduction in both gambling behavior and urges on a mean dosage of 157 mg/ day of naltrexone. In an 11-week double-blind, placebo-controlled study [50], 83 patients with PG were randomly assigned to naltrexone (up to 250 mg/day, mean final dosage of 188 mg/day), or to placebo. Of the 45 completers receiving naltrexone, 75% showed improvement on the outcome measures (patient- and clinician-rated CGI), compared to 24% of patients receiving placebo. The authors reported that depressive symptoms were mild or absent in the completers receiving naltrexone. Furthermore, patients with moderate or higher levels of gambling urges at baseline had a better response to naltrexone than other patients. It is possible to argue that urges, which represent one of the core symptoms of PG, might also be the main target of naltrexone. As suggested by Kim's group, baseline urge level might be used as a stratification variable that could enhance group differences in outcome and could also be used to predict response to naltrexone. However, the side-effect profile of this compound may be problematic. Since higher naltrexone doses are needed in PG treatment than are usually employed for alcohol and drug dependence, the increase in transaminase levels must be carefully monitored due to the risk of hepatotoxicity. In addition, other common side-effects reduce patient compliance. Currently, research is being conducted on another opioid receptor antagonist, nalmefene, to assess its efficacy in treating pathological gambling with preliminary encouraging findings.

### Mood Stabilizers (see Table 5 - additional file: 1)

Evidence of the effectiveness of mood stabilizers in ICDs has been reported [77-85], suggesting these compounds can modify, and treat successfully, some core features of these disorders. In 1980, Moskowitz [86] reported the effectiveness of lithium in treating 3 pathological gamblers with bipolar features. In 1994, Haller and Hinterhuber reported, in a placebo controlled studyl [87], a single case of chronic PG successfully treated with carbamazepine at a dosage of 600 mg/day. Subsequently, a single-blind placebo-controlled study [88] was conducted by our group in order to evaluate the efficacy and safety of lithium and valproate in non-bipolar pathological gamblers. At the end of the 14th week, 14 of the 23 patients receiving lithium (mean dose 795 mg/day) and 11 of the 19 taking valproate (mean dose 870 mg/ day) were considered responders (PG-CGI and PG-YBOCS). Although patients with bipolar disorder were excluded from thus study as well as patients with current alcohol/drug addiction or schizophrenic spectrum disorders, the majority of patients had a past history of other psychiatric conditions, including depressive episodes (7/45), alcohol or drug abuse (23/45), panic disorder (15/45), OCD (9/45), antisocial personality disorder (8/45) and other ICDs (16/45). Therefore, even if a specific "anti-impulsive" action of mood stabilizers may be hypothesized, we have to consider the impact of lifetime comorbidities, which may currently be subthreshold, when considering treatment-response data. In addition, although the Structured Clinical Interview for DSM-IV (SCID) [89] was used in the study to exclude the comorbid bipolar disorders, the accuracy of this instrument in detecting bipolar disorder, particularly type II, is still matter of debate. Consequently, the possible inclusion of bipolar II patients in the study should be kept in mind.

Recently, our group conducted the first placebo-controlled treatment study [90] of sustained-release lithium carbonate in pathological gamblers with bipolar spectrum disorders. Patients with bipolar I disorder were excluded because it was a placebo-controlled trial, but patients with diagnoses of bipolar II, bipolar disorder NOS and cyclothymia were included in the trial. Among the 29 completers at the end of the 10^th ^week, 12 patients received lithium (mean dose 1150 mg/day) and 17 received placebo. At endpoint, 10/12 patients in the lithium group were considered responders (based on CGI of 1 or 2 and a score reduction of 35% on the PG-YBOCS), and improvement in impulsive gambling significantly correlated with increases in affective stability. Of note, the percentage of placebo responders found in this study (29%) appears to be significantly lower than that reported in other studies. This study, deliberately designed to evaluate the effectiveness of a mood stabilizer in pathological gamblers with comorbid bipolar spectrum features confirms previous reports of the effectiveness of these compounds in PG, and suggests that there may be significant advantages in subtyping pathological gamblers. The identification of bipolar spectrum PG patients is relevant to the choice of pharmacological treatment; in this subtype, in fact, a mood stabilizer might have a higher probability of efficacy than other treatments such as SSRIs, which may exacerbate affective instability and cause a gambling relapse.

Recently, Dannon and his group randomized 31 male, pathological gamblers to topiramate or fluvoxamine (both titrated up to 200 mg/day) in a 12 week blind-rater comparison trial [91]. At endpoint, both groups reported improvement on the PG-CGI, although the improvement for fluvoxamine did not quite reach statistical significance (p < 0.08 for fluvoxamine; p < 0.01 for topiramate); there were no statistically significant differences between the 2 groups. In addition, a larger number of drop-outs were reported in the fluvoxamine group. Notwithstanding the lack of a placebo-controlled group and the partial blind design of this trial, it is noteworthy that a mood stabilizer was at least as effective as an SSRI in a group of pathological gamblers without comorbid conditions, providing, therefore, more than one choice for this specific subgroup of gamblers.

Among the 18 studies reported, 6 were double-blind, placebo-controlled studies (4 with SRIs, 1 with naltrexone and 1 with a mood stabilizer), 3 were single-blind (1 with an SRI and 1 comparing mood stabilizers, and one comparing an SRI with a mood stabilizer), 4 were open-label (1 with an SRI, 1 with nefazadone, 1 with naltrexone, and one with bupropion) and 4 were case-reports (2 with naltrexone and 2 with mood stabilizers, one of which had a double blind design).

With regard to reported SRI trials, a total of 127/200 completed single- and double-blind trials (41 with fluvoxamine and 86 with paroxetine), 15 patients completed an open-label trial with citalopram (see Tables 1 and 2 - additional file: 1). In addition, 12 patients received open-label treatment with nefazodone and 10 patients with bupropion (Table 3 - additional file: 1).

Thirty-six out of forty-five patients completed a double-blind trial with naltrexone, 14/17 patients completed an open-label trial (table 4 - additional file: 1), and 2 patients were reported in 2 different case reports to have received open label treatment.

Seventy-two out of ninty-seven patients completed single- and double-blind trials with mood stabilizers (27 with lithium, 16 with valproate, 12 with topiramate and 17 treated with placebo Table 5), and 4 cases of patients treated with mood stabilizers were reported, one of which treated in a double-blind design.

The majority of pathological gamblers have been treated with SRIs, and these drugs seem to be generally well tolerated. Effective does in PG resemble those used in the treatment of OCD; approximately 200 mg/day for fluvoxamine, and up to 60 mg/day for paroxetine were effective in PG trials. These studies demonstrated a marked placebo effect, and therefore they need an adequate period of time (between 8 and 12 weeks) in order to monitor and assess the improvement achieved. Only one SRI trial [44] looked at the relationship between worsening PG symptoms in non-responders and comorbid cyclothymia. In all the studies, the efficacy of the SRIs, when statistically significant, was independent of underlying depressive or anxious comorbidity. This supports the hypothesis that a serotonin sensitive PG subtype exists in which the marked impulsivity is driven by serotonergic dysregulation. In this subtype, the use of an SRI might normalize the 5-HT dysfunction and improve the clinical condition. An important exception, however, is the subgroup of pathological gamblers with bipolar features, for whom treatment with an SRI could precipitate worsening of the overall clinical picture. Therefore, although SRIs have been shown to be effective in patients with mild obsessive-compulsive, depressive and anxious comorbidity, additional research should be conducted on PG cormorbid with bipolar spectrum disorders. In this research, it would be important to consider not only current comorbid diagnoses but also the past history of the patient and careful attention to other current symptoms to assess the presence of subthreshold bipolar psychopathology [88]. Future double-blind studies are necessary in order to acquire further and more detailed information about the efficacy of the SRIs already employed in the treatment of PG, as well as of those which have never been tested.

Although only 1 double-blind study has been conducted in PG with naltrexone, its results may have important implications. The target of this opioid antagonist seems to be the urge to gamble. Even if this core symptom is also a feature of bipolar disorder, it is sensitive to the action of naltrexone, and its predominance in specific cases might indicate this opioid antagonist should be considered. In addition, the presence of comorbid substance and alcohol abuse/dependence might also suggest that naltrexone should be tried, as shown by the case reports. However, although naltrexone has demonstrated specific anti-impulsive properties, it could be difficult to consider this drug a first-choice treatment for PG, given the high dosage required to be effective, its potential hepatotoxicity risk and the side-effects profile reported. However, pathological gamblers with specific comorbid profiles and without hepatic diseases may be successfully treated with this opioid antagonist. Finally, promising preliminary findings [92] have been shown with another opioid antagonist, nalmefene, which may have better tolerability than naltrexone.

The studies conducted with mood stabilizers confirm what has been postulated about the SRIs. A specific anti-impulsive property of mood stabilizers can be hypothesized. In addition, the impulsivity may be related to affective instability and, therefore, a decrease in affective instability with mood stabilizers might reduce impulsivity, improving the overall clinical condition. The findings support the hypothesis of a PG subtype in which the administration of SRIs would be useless or even worsen the associated bipolar spectrum symptoms. Specific contraindications for the use of mood-stabilizers in PG have not yet been reported. Although additional confirming research is necessary, the possible efficacy of these compounds in a wide spectrum of pathological gamblers was recently supported by an open-label study in which fluvoxamine and topiramate showed similar efficacy in treating PG.

The most recent double-blind PG study reported by our group [92] included only PG patients with comorbid bipolar II, cyclothymia and bipolar NOS diagnoses. The recognition of this as a possibly important subgroup of PG led to this study of the efficacy of lithum in gamblers with PG and bipolar spectrum disorders. These patients demonstrated statistically significant improvement compared to placebo on all key outcome measures, with 83% of the patients on lithium considered responders compared to 29% on placebo, a notably smaller placebo effect than normally reported in SRIs trials. The inclusion of adequate clinical instruments and interviews in order to obtain precise comorbid diagnoses may enable complex findings to be interpreted and lead to more precisely targeted treatments. Only a minority of studies have done this. Generally authors prefer to select samples of patients without other current psychopathology, in order to obtain samples that are as homogeneous as possible and to have a sample of "pure" PG patients. However, this procedure, though excluding complicating comorbid pathologies, does not exclude the possible influence of the subthreshold psychopathology, which may influence the treatment-response. In addition, such a procedure may lead to treatments which are effective for only a small and atypical group of pathological gamblers.

Future studies are needed in order to confirm the clinical validity of dividing pathological gamblers into specific subgroups. For example, neuroimaging studies might assess if different subgroups of patients show common patterns of neuronal activation, metabolism and perfusion of specific brain areas.

## Conclusion

Treatment data supports the hypothesis that there are specific subgroups of pathological gamblers. These subgroups were suggested by the different models of PG that were discussed. The assessment of clinical and subclinical comorbid psychopathology seems to represent a rational and valid approach to selecting pharmacological treatment for the different subgroups. However, few studies reported to date have specifically explored the comorbid profile of pathological gamblers. Nevertheless, positive findings shown in some studies and interesting observations reported in other studies, suggest this approach support is promising and might lead to more effective treatments for specific subgroups of gamblers.

## Competing interests

Disclosure: Dr. Dell'Osso and Dr. Allen do not have an affiliation or financial interest in any organization that might pose a conflict of interest. Prof. Hollander is on the advisory board of Abbott, Ortho-McNeil and Solvay.

## Supplementary Material

Additional file 1 Click here for file
