# Supplementary material for: Comorbidity issues in the pharmacological treatment of pathological gambling: a critical review
Source: Clin Pract Epidemiol Ment Health. 2005 Oct 10;1:21. doi: 10.1186/1745-0179-1-21 (PMC1283978; doi:10.1186/1745-0179-1-21)
Supplement: Additional file 1 [file 1745-0179-1-21-s1.doc]

**Table 1. SSRIs: double-blind, placebo-controlled studies in pathological gambling**

| Citation | Study Design & Sponsor | Sample features | Tx group | Control Group | Trial Length | Outcomes | Conclusions |
| --- | --- | --- | --- | --- | --- | --- | --- |
| Blanco et al 2002 | RA;DB;  PC;N-ITT | 32 pathological gamblers,  13 completers at 6 months | **Fluvoxamine** (FLV) up to 200 mg/day  N=15  3 drop-outs due to side-effects | Placebo  N=17  1 drop-out due to side-effects | 6 months | No significant differences in the proportion of responders between patients treated with FLV and those assigned to Pl for the overall trial | Small sample study showing no statistically significantly difference of FLV from PL except in males and younger patients |
| Hollander et al 2000 | RA;DB;  PC;N-ITT | 15 pathological gamblers,  10 completers | **Fluvoxamine** (FLV) up to 250 mg/day, final mean dose 195 + 50 mg/day  N=15  3 drop-outs due to side-effects | Placebo | 8 weeks  (12 weeks) | 75% of the pts were judged treatment responders (PG-CGI) to FLV I phase I and 67% in phase II, as compared with 67% responders to Pl in phase I and 25% in phase II | Small sample, crossover study showing the effectiveness of FLV in PG. Post hoc analysis, treating each phase as a separate trial, demonstrated a significant difference between FLV and Pl in the 2^nd^ phase of the trial but not in the 1^st^ phase |
| Kim et al 2002 | RA;DB;  PC;ITT | 45 pathological gamblers,  41 completers | **Paroxetine** (PAR) up to 60 mg/day  N=23  1 drop-out due to side-effects | Placebo  N=22  1 drop-out due to side-effects | 8 weeks | At the endpoint, the mean G-SAS total had decreased by 52% in the PAR group compared with 23% in the Pl group. CGI-rates showed more than 60% of responders in the PAR group compared to less than 25% in the Pl group | Small sample study showing the superiority of PAR over Pl in treating acute PG |
| Grant et al 2000 | RA;DB; PC;ITT | 76 pathological gamblers,  45 completers | **Paroxetine** (PAR) up to 60 mg/day  N=36  6 drop-outs due to side-effects | Placebo  N=40  1 drop-out due to side-effects | 16 weeks | Treatment with PAR did not yield significantly greater efficacy than placebo at study endpoint as assessed by the PG-CGI | Multicenter trial showing no evidence of statistically significant advantage for paroxetine on any of the outcomes measures |

RA= random assignment; DB= double-blind; SB= single-blind; UB= unblinded; PC= placebo controlled; UC= uncontrolled; ITT= intent-to-treat analysis

N-ITT= no intent-to-treat analysis

**Table 2. SSRIs: single-blind, placebo-controlled and open-label studies in pathological gambling**

| Citation | Study Design | Sample features | Tx group | Control Group | Trial Length | Outcomes | Conclusions |
| --- | --- | --- | --- | --- | --- | --- | --- |
| Hollander et al 1998 | NRA;SB;PC;N-ITT | 16 pathological gamblers,  10 completers, no drop-out due to side-effects | **Fluvoxamine** (FLV) up to 300 mg/day  Mean dose 220 mg/day | Placebo  (Before receiving FLV, patients entered an 8-week lead in phase) | 16 weeks | At endpoint 7/10 patients were judged responders (much or very much improved on CGI and decrease < 25% on PG-Y-BOCS) | Small sample study showing the effectiveness of FLV in treating acute pathological gambling |
| Zimmerman et al 2002 | NRA;UB;  UC;N-ITT | 15 pathological gamblers,  8 of these with major depression,  9 completers,  1 drop-out due to side-effects | **Citalopram** (CIT) up to 60 mg/day  Mean final dose 34.7 mg/day |  | 12 weeks | 13/15 10 patients were judged responders (much or very much improved on CGI; ΔPG-Y-BOCS 79.5%) | Small open-label trial showing the efficacy of CIT in treating acute pathological gambling |

RA= random assignment; DB= double-blind; SB= single-blind; UB= unblinded; PC= placebo controlled; UC= uncontrolled; N-ITT= no intent-to-treat analysis

**Table 3. Other antidepressants (non SSRIs): open-label studies in pathological gambling**

| Citation | Study Design | Sample features | Tx group | Control Group | Trial Length | Outcomes | Conclusions |
| --- | --- | --- | --- | --- | --- | --- | --- |
| Pallanti et al 2002 | NRA;UB;  UC;N-ITT | 14 pathological, gamblers  12 completers,  no drop-out due to side-effects | **Nefazodone** (NEF) up to 500 mg/day  Mean dose 345.8 mg/day |  | 8 weeks | 9/12 completers were rated as responders on the basis of both PG-CGI score of 1 or 2 and a 25% reduction in PG-Y-BOCS score | Small open-label study showing the effectiveness of NEF in treating acute pathological gambling |
| Black et al 2004 | NRA;UB;  UC;N-ITT | 10 pathological gamblers | **Bupropion** (BUP) up to 300 mg/day |  | 8 weeks | 7/10 patients were rated as responders on the basis of a PG-CGI score of 1 or 2 | Small open-label study showing the effectiveness of BUP in treating acute pathological gambling |

RA= random assignment; DB= double-blind; SB= single-blind; UB= unblinded; PC= placebo controlled; UC= uncontrolled; N-ITT= no intent-to-treat analysis

**Table 4. Opioid antagonists: double-blind, placebo-controlled and open-label studies in pathological gambling**

| Citation | Study Design | Sample features | Tx group | Control Group | Trial Length | Outcomes | Conclusions |
| --- | --- | --- | --- | --- | --- | --- | --- |
| Kim et al 2001 | RA;DB;  PC;ITT | 45 pathological gamblers,  36 completers,  4 pts developed elevated liver transaminases | **Naltrexone** (NLT) N=20, up to 250 mg/day  Mean dose 187 mg/day | Placebo  N=25 | 12 weeks | At endpoint NLT showed significant improvement over the placebo group in all measures including PG-CGI | The only DB, PC study with NLT showed the effectiveness of this opioid antagonist in treating pathological gambling. Besides those pts excluded during the enrollment, 4 other subjects developed elevated liver transaminases during the study period |
| Grant et al 2001 | NRA;UB;  UC;NITT | 17 pathological gamblers,  14 completers,  2 drop-outs due to side-effects | **Naltrexone** (NLT) up to 250 mg/day  Mean final dose 157 mg/day |  | 6 weeks | At endpoint, most patients had stopped their gambling behavior and reported significant decreases in the CGI and other gambling symptoms | Small open-label trial showing the efficacy of NLT in treating acute pathological gambling |

RA= random assignment; DB= double-blind; SB= single-blind; UB= unblinded; PC= placebo controlled; UC= uncontrolled; ITT= intent-to-treat analysis

N-ITT= no intent-to-treat analysis

**Table 5. Mood stabilizers: randomized, placebo-controlled and active-comparison studies in pathological gambling**

| Citation | Study Design | Sample features | Tx group | Control Group | Trial Length | Outcomes | Conclusions |
| --- | --- | --- | --- | --- | --- | --- | --- |
| Hollander et al 2005 | RA;DB;  PC;ITT | 40 pathological gamblers with comorbid bipolar II, bipolar NOS or cyclothymia),  29 completers (12 LI compl, no drop-out due to side-effects and 17 Pl compl, no drop-out due to side-effects) | **Lithium** (LI) up to 1200 mg/day, Mean final dose 1150 + 215 mg/day  N=18 | Placebo  N=22 | 10 weeks | Gambling severity was statistically significantly lower in the LI group than in the Pl group at endpoint based on the PG-YBOCS and PG-CGI scores. Significant improvements in mood instability scores on the CARS-M were also noted in the LI group vs the Pl group | Small double-blind study the efficacy of lithium in treating pathological gamblers with bipolar spectrum comorbidity |
| Pallanti et al 2002 | RA;SB;  AC;ITT | 42 pathological gamblers,  31 completers (15 LI compl, 2 drop-outs due to side-effects and 16 VAL compl, 1 drop-out due to side-effects) | **Lithium** (LI) up to 1200 mg/day;  N=23  Mean final dose 795 + 261 mg/day | **Valproate** (VAL) up to 1500 mg/day  N=19  Mean final dose 830 + 280 mg/day | 14 weeks | At the endpoint both groups both groups showed significant mean percentage improvement on PG-YBOCS score, but the improvement difference between groups was not statistically significant | Small single-blind study showing that both mood-stabilizers (LI and VAL) were effective in the treatment of pathological gambling |
| Dannon et al 2005 | RA;AC;  NITT;  Blind raters | 31 pathological gamblers,  20 completers (12 TOP compl, 2 drop-outs due to side-effects and 8 FLV compl, 5 drop-outs due to side-effects) | **Topiramate** (TOP) up to 200 mg/day;  N=15 | **Fluvoxamine** (FLV) up to 200 mg/day  N=16 | 12 weeks | At the endpoint both groups both groups showed significant mean percentage improvement on PG-CGI score, but the improvement difference between groups was not statistically significant | Small blind-rater comparison study showing that the mood-stabilizers TOP and the SSRI FLV were equally effective in the treatment of pathological gambling |

RA= random assignment; DB= double-blind; SB= single-blind; UB= unblinded; PC= placebo controlled; AC= active-comparison; UC= uncontrolled; ITT= intent-to-treat analysis
